# Synthesis of a Cleaved Form of Osteopontin by THP-1 Cells and Its Alteration by Phorbol 12-Myristate 13-Acetate and BCG Infection

**DOI:** 10.3390/ijms19020418

**Published:** 2018-01-31

**Authors:** Gaowa Bai, Hirotoshi Motoda, Ryo Ozuru, Haorile Chagan-Yasutan, Toshio Hattori, Takashi Matsuba

**Affiliations:** 1 Department of Health Science and Social Welfare, Kibi International University, 8 Igamachi, Takahashi 716-8508, Japan; gaowabai@kiui.ac.jp (G.B.); hmotoda@kiui.ac.jp (H.M.); haorile@foxmail.com (H.C.-Y.); 2 Division of Bacteriology, Department of Microbiology and Immunology, Faculty of Medicine, Tottori University, Yonago, Tottori 683-8503, Japan; ozuru@med.tottori-u.ac.jp; 3 Mongolian Psychosomatic Medicine Department, International Mongolian Medicine Hospital of Inner Mongolia, Huhhot 010065, China

**Keywords:** osteopontin, THP-1, protease, BCG, tuberculosis

## Abstract

The protease-cleaved osteopontin (OPN) was proposed to enhance the migration of memory T cells to granulomas in tuberculosis. Various forms of OPN were identified in human monocytic THP-1 cells stimulated by phorbol 12-myristate 13-acetate (PMA). Antibodies O-17, 10A16 and 34E3, which recognize N-terminus, the C-half, and thrombin-cleaved site of OPN, respectively, all detected distinct bands on Western blots following PMA stimulation. Bands corresponding to 18 and 30 kD were detected by antibodies 34E3 and 10A16, indicating that OPN cleavage occurred by endogenous proteases in the PMA-stimulated THP-1 cells. In immune-fluorescence (IF) assay, 34E3 positive signals were detected in intracellular space of non-infected and bacillus Calmette-Guérin (BCG)-infected cells; however, 10A16 positive signals were confirmed in extracellular area in PMA-stimulated cells followed by BCG infection. Small amounts of full-length (FL) and thrombin-cleaved (Tr) OPN were detected by ELISA in the supernatants of non-PMA-stimulated cells, and increased levels of all forms, including undefined (Ud) OPN, in PMA-stimulated cells. ELISA showed a decrease in OPN synthesis during BCG infection. To our knowledge, this is the first report of OPN cleavage in THP-1 macrophages after PMA stimulation, and of enhanced cleavage induced by BCG infection.

## 1. Introduction

Matricellular proteins (MCPs) constitute a family of secreted extracellular matrix (ECM) proteins that influence cell–matrix interactions [1]. Based on this definition, several proteins have been identified as MCPs, including connective-tissue growth factors, galectins [2] and osteopontin (OPN) [3]. MCPs participate in wound repair, inflammation and cancer progression by binding to their receptors, with OPN binding to integrins and CD44 variants [3]. The multifunctional aspects of MCPs are derived from the different structural proteins, cell-surface receptors, proteases and cytokines with which these proteins come into contact in the local environments of various tissues. 

*Mycobacterium tuberculosis* (MTB) evades the host immune system by various mechanisms including inhibition of phagolysosome fusion within phagocytes or induction of anti-inflammatory cytokine secretion [4]. Abnormal turnover of MCPs in the development of granulomas and cavities are the typical pulmonary manifestations of tuberculosis (TB) [5], in which chronic inflammation is activated, leading to tissue damage and subsequent tissue remodeling [6]. In a previous study, we observed the expression of OPN and Gal-9 in TB granuloma [7]. We also confirmed a high level of plasma OPN in subjects with MTB from the Philippines [8] and from Indonesia [7]. Full-length OPN (FL-OPN), the intact form of OPN, is involved in the complex pathways of coagulation and fibrinolysis, where multiple sites of FL-OPN serve as targets for protease(s) cleavage. During this process, OPN fragments are produced. Among those fragments, proteolytic cleavage of FL-OPN by thrombin (between Arg168 and Ser169) generates a functional fragment of N-terminal thrombin-cleaved OPN (trOPN), which contains a cryptic binding site for integrins α9β1 and α4β1 that enhances the attachment of trOPN to integrins. Increases in trOPN levels have been reported in the recovery phase of dengue virus (DENV) infection [9]. Furthermore, other OPN forms are detected in DENV infections using a different ELISA system, which include a mixture of FL-OPN, trOPN and undefined OPN (Ud-OPN) [9].

Higher plasma concentrations of Ud-OPN, but not FL-OPN or trOPN, negatively correlate with TB-specific memory T-cell numbers represented by interferon γ (IFN-γ)-secreting cell numbers of ESAT-6-stimulated peripheral blood lymphocytes [10]. The levels also closely correlate with its receptor, the soluble form of CD44 (sCD44) [10].

It is also known that other enzymes such as matrix metalloproteinases (MMPs) can cleave OPN at sites other than the thrombin cleavage sites [11,12]. Accumulation of α4β1 and other integrin-bearing cells are reported in MTB infection [13]. Furthermore, the osteopontin is subject to genetic variation, and variants of the *OPN* gene including single-nucleotide polymorphisms (SNPs) and alternative splicing, could contribute to the development and/or progression of specific cancers. [14,15]. These findings led us to study the expression of different OPN forms using PMA-stimulated monocyte-derived cells, and to observe the effects of bacillus Calmette-Guérin (BCG) infection on the alteration of their production.

## 2. Results

### 2.1. Western Blot

**Figure 1:**
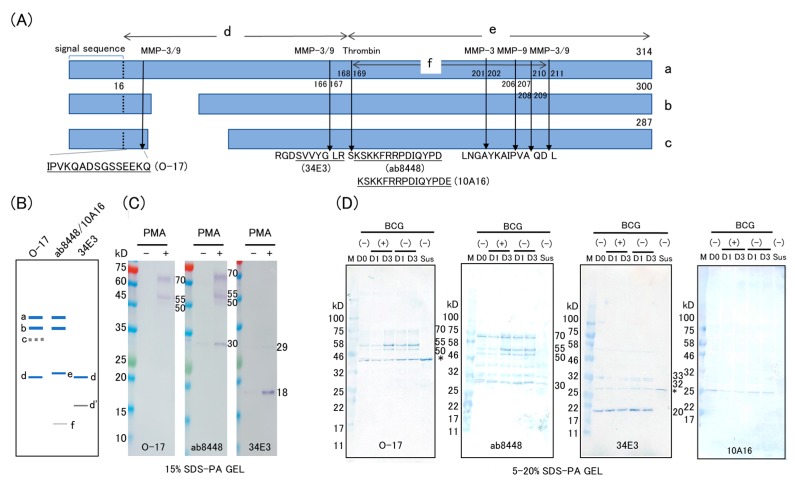
Western blot analysis of lysates from *M. bovis* BCG-infected THP-1 cells stimulated with PMA. (**A**) Schematic of OPN isoforms (a–c) with MMP 3, MMP 9, and thrombin cleavage sites (arrows) and antibody epitopes for antibodies O-17, 34E3, ab8448 and 10A16 used for Western blotting. Three isoform structures of human OPN [14,15] and the known epitope of the antibodies used for Western blotting are shown. The MMPs cleavage sites are predicted by Protease specificity prediction server (Prosper, https://prosper.erc.monash.edu.au/); (**B**) the expected sizes of the OPN products recognized by each antibody by Western blotting are diagrammatically shown; (**C**) western blot analysis of PMA-stimulated and non-stimulated THP-1 cells. Cells were cultured with 10 ng/mL of PMA (16.2 nM) for 2 days and the cell lysates were subjected to SDS-PAGE (15%) followed by immunostaining using an immunodetection kit; (**D**) western blot analysis of BCG-infected THP-1 cells (5–20% gradient SDS-PAGE) developed using a peroxidase substrate system. PMA-stimulated cells (D0) were infected with BCG for 1 day (D1), and 3 days (D3). Non-stimulated cells (Sus) served as controls. Asterisks indicate nonspecific band.

Four antibodies that identify different epitopes of OPN were used in this study. The schematic structure of human OPN isomers and their predicted thrombin fragments are shown in Figure 1A,B. Polyclonal rabbit antibody O-17 is specific to the N-terminus of OPN (Ile17–Gln31), and anti-trOPN monoclonal antibody 34E3 is specific to the epitope Ser162–Arg168, which is exposed by thrombin digestion [9,16]. Mouse monoclonal antibody 10A16 and polyclonal rabbit antibody ab8448 were generated against synthetic peptides corresponding to the human OPN internal sequences Lys166–Glu187 and Ser165–Asn186, respectively. Cleavage sites for MMPs and the predicted fragment sizes of OPN isoforms [11,14,15] are also depicted. None of the antibodies detected distinct bands in cell lysates without PMA treatment, except for a very faint 30-kD band with ab8448 (Figure 1C), corresponding to the C-half of OPN (product e). After PMA stimulation, both the antibodies O-17 and ab8448 detected FL-OPN (product a, Figure 1A; 70-kD band, Figure 1C), and a 68-kD band (product a), as well as smaller fragments (product b in Figure 1A; 55 kD in Figure 1C). Furthermore, a distinct band corresponding to C-half of OPN (30 kD in Figure 1C,E) was detected by ab8448 after PMA stimulation. Antibody O-17, which is specific to the N-terminus OPN, failed to recognize the counterpart of the cleaved product (trOPN, 27 kD, product d). Antibody 34E3, which is specific to the thrombin-cleaved site, detected a very faint band corresponding to trOPN (33 kD in Figure 1C) and a distinct novel band of 18 kD (Figure 1C). The 18-kD band was not recognized by O-17, probably due to the lack of the N-terminus epitope. Antibody 10A16 failed to detect either product a, or product b, and only labeled a distinctive band corresponding to product e (C-half of OPN). After BCG infection, no additional bands were detected, but apparent reduction of the full-length OPN was observed (Figure 1D).

### 2.2. Immunofluorescence Study

#### 2.2.1. Single-Color Study of PMA-Stimulated Cells 

**Figure 2:**
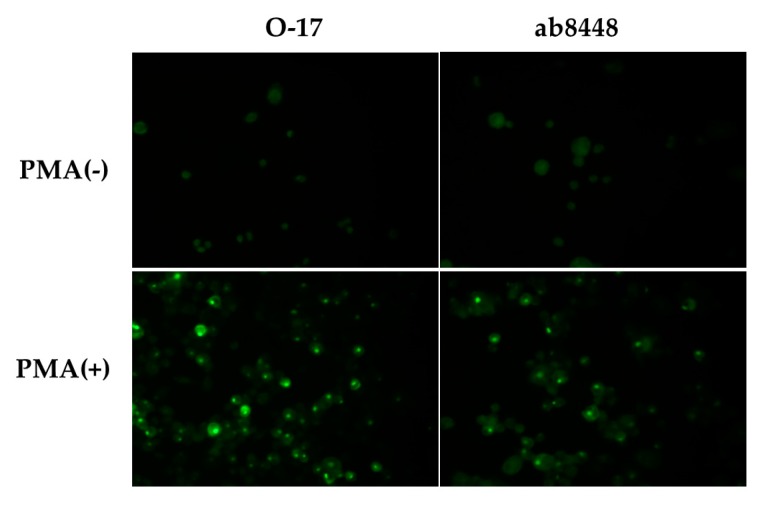
Immunofluorescence study of PMA-stimulated THP-1 cells. Cells were cultured for 2 days with PMA (**bottom**) or without PMA (**upper**). The cells were stained with anti-OPN antibodies O-17 or ab8448. An Olympus fluorescence microscope was used for the analysis. (Original magnification ×400).

PMA-stimulated and non-stimulated cells were examined for the expression of OPN. Antibody O-17 showed a very weak, diffuse staining in non-stimulated cells, but distinct dot-like positive staining cells were observed after PMA stimulation. Similarly, clear signals were seen with ab8448 following PMA stimulation (Figure 2).

#### 2.2.2. Multicolor Analysis

**Figure 3:**
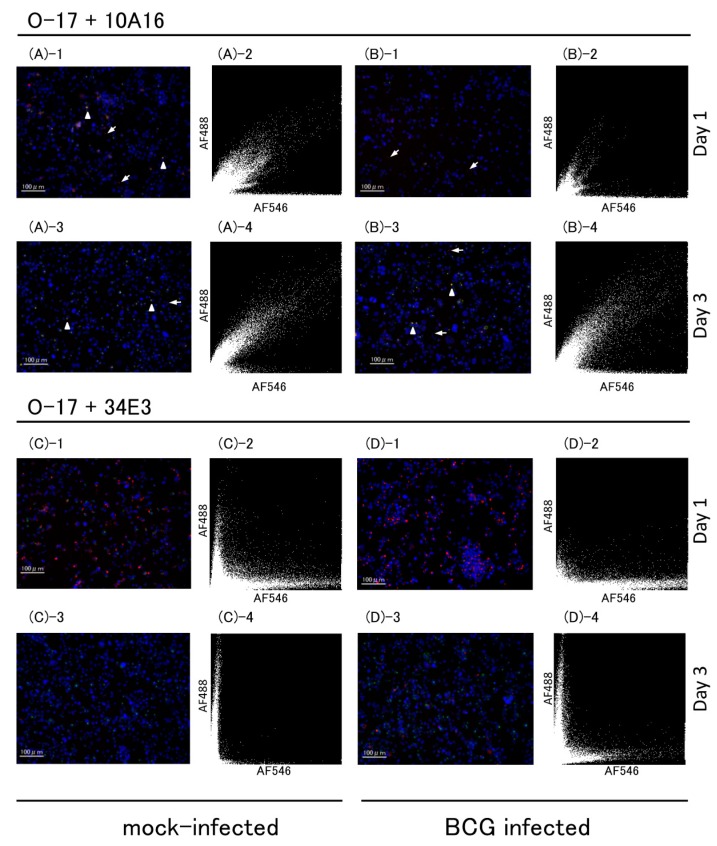
Multicolor immunofluorescence of BCG-infected THP-1 cells. BCG- or mock-infected cells were immuno-stained using three anti-OPN antibodies, (O-17, 34E3 and 10A16). Multicolor immunofluorescence images (left) and scattergrams (right) from mock-infected (**A**,**C**) and BCG infected cells (**B**,**D**). The scattergram was drawn based on fluorescence image on the left of each. *X* axis indicate fluorescence intensity of 10A16 (**upper**) and 34E3 (**lower**), respectively. *Y*-axis indicates that of O-17. Arrows indicate 10A16 and arrow heads indicate co-localization of 10A17 and O-17. Micrograph scale bars represent 100 µm. (Original magnification ×200).

**Figure 4:**
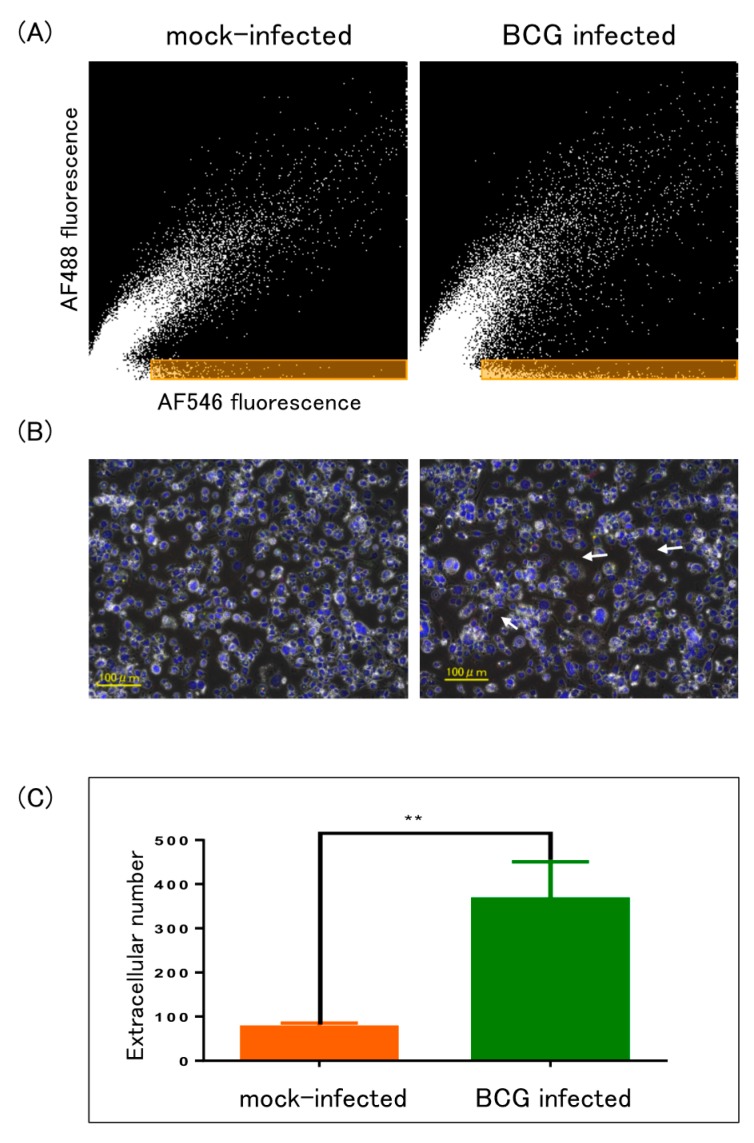
Enumeration of extracellular signals. Fluorescence intensity of O-17 and 10A16 were analyzed by Hybrid cell count module. (**A**) Extracellular signals were highlighted in orange area of scattergrams drawn based on Figure 3(A-4, B-4), respectively. *X*-axis and *Y*-axis indicate 10A16 and O-17 fluorescence intensity, respectively; (**B**) phase contrast image was overlaid onto fluorescent image of Figure 3(A-3, B-3), respectively. Arrows indicate apparent extracellular signals of 10A16; (**C**) the extracted numbers of extracellular 10A16 signals in orange area of BCG-infected and mock-infected cells were compared. ** *p* < 0.01, *t* test done by prism software.

Multicolor immunofluorescent analysis for OPN was also conducted using BCG-infected THP-1 cells. Figure 3 shows fluorescent microscopic images and the scattergrams from the images. Both in mock-infected and BCG-infected cells, double-positive signals of 10A16 and O-17 were detected in intracellular area (arrow heads); however, single-positive signals of 10A16 were detected at apparent extracellular area (Figure 3(A-1,A-3,B-1,B-3), arrows). Number of O-17 positive signals were low in BCG-infected cells at day 1 and increased at day 3 (Figure 3(B-2,D-2) vs. Figure 3(B-4,D-4)). The double-positive cells increased at day 3 in BCG-infected cells (Figure 3(B-4)). 34E3 positive signals in both mock-infected and BCG-infected cells were detected only in intracellular area and the positive cells apparently decreased at day 3. Double-positive signals of O-17 and 34E3 were few even in scattergrams (Figure 3(C-2,D-2)). 34E3 positive signals are few in mock-infected cells but still detected at day 3 of infected cells (Figure 3(C-3,C-4) vs. Figure 3(D-3,D-4)). 

The apparent extracellular signals of 10A16 in mock-infected and BCG-infected cells at day 3 were further analyzed using Hybrid cell count module. The signals of 10A16 were highlighted in orange area (Figure 4A) and by arrows on the merged image (Figure 4B, right panel), and the extracted numbers were compared (Figure 4C). Significantly higher numbers were extracted by BCG-infected cells than by mock-infected cells.

### 2.3. ELISA

**Figure 5:**
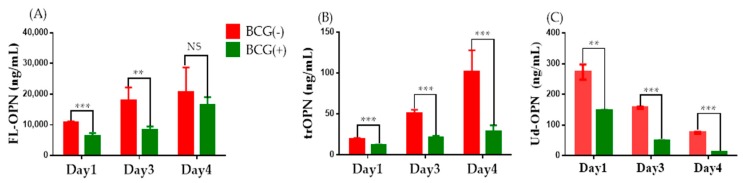
OPNs in BCG-infected THP-1 cells. PMA-stimulated THP-1 cells were infected with BCG and the OPNs were measured in the supernatants on the day post-infection indicated. (**A**) Full-length OPN (FL-OPN); (**B**) Thrombin-cleaved OPN (trOPN); (**C**) Undefined OPN (Ud-OPN). ** *p* < 0.01, *** *p* < 0.001, Multiple *t* test done by prism software.

To know if the apparent extracellular signals are associated to the amounts of OPN in culture supernatants (CS), three different ELISA kits were used to characterize the OPN in PMA-stimulated, BCG-infected, or mock-treated cells. Small amounts of FL-OPN and trOPN were detected in culture supernatants but no Ud-OPN was detected. Following PMA simulation, higher amounts of OPN were detected in all three assays (Table 1). PMA-stimulated cells were subjected to BCG infection, and their OPN production was observed for four days. Both FL-OPN and trOPN levels increased each day, but BCG-infected cells produced significantly lesser OPN than the mock-infected control cells. In contrast, Ud-OPN levels gradually decreased each day over the four days monitored. The Ud-OPN levels in BCG-infected cells similarly decreased compared with those in the non-infected cells (Figure 5).

## 3. Discussion

To our knowledge, this is the first report to characterize OPN expression in THP-1 cells, a human monocyte-derived cell line, using antibodies against distinct epitopes of the protein. Because OPN has been reported to have a pivotal role in tuberculosis infection, the effect of BCG infection on the expression by THP-1 cells was also studied. OPN is known to be involved with tumor-associated macrophages (TAMs) [17,18]. Moreover, the proinflammatory functions of OPN from bone marrow-derived macrophages using OPN-deficient mice have been described [19]. Identification of OPN protein in THP-1 cells has not been described, although gene expression of OPN by THP-1 cells is reported [20]. Western blot analysis using ab8448 detected a faint band of approximately 30 kD in non-stimulated THP-1 cells. Antibodies O-17 and ab8448 both demonstrated only minimal signals for OPN by immunofluorescence. With ELISA using O-17 and 10A16, a small amount of FL-OPN and trOPN were detected. These findings are consistent with the constitutive expression of OPN, and accounts for the presence of the 30-kD band, corresponding to C-half of OPN, suggesting that endogenous proteases of the cells can cleave OPN. It is known that exposure of THP-1 cells to PMA increases OPN mRNA expression and protein levels in a time-dependent manner [20]. Immunofluorescence analysis of the PMA-stimulated THP-1 cells with ab8448 gave a clear punctate positive finding. Similar dot-like findings were previously reported in multinucleated giant cells of TB granulomas [21]. Based on Western blotting, we clearly detected cleaved OPN products, as well as the full-length product. It is not clear why O-17 failed to detect any cleaved products, which were detected by 34E3. Notably, 34E3 detected a novel 18-kD band. Because 34E3 is known to be specific for the thrombin-cleaved site SVVYGLR [14], the trOPN fragment is expected to be 32 kD. The finding of a new, shorter 18-kD form by 15% SDS-PAGE, and a 20-kD form by 5–20% gradient SDS-PAGE, suggests that the trOPN may be further processed by other mechanisms. Analysis of multiple cleaved sites of OPN derived from milk also shows a smaller fragment of similar size following thrombin digestion [12]. However, in the current study, we were able to detect the band without the need of any artificial digestion. MMP-9 is the highest induced protein in PMA-treated THP-1 cells, and its activities may be responsible for the cleavage [22].

It is also known that a small peptide corresponding to the cleaved site binds to the α4 integrin with a similar affinity as the LDV peptide [23]. Therefore, the novel 18-kD band may have significant integrin binding activity. These activities may be related with the involvement of very late antigen (α4β1 integrin) expressing immune cells (T cells and macrophage) in tuberculoma, which are detected in a macaque model by positron emission tomography [13]. MTB infection in THP-1 cells causes activation of MMP-9 through micro RNA-206 [24] and alters cathepsin activities in cells [25]. Activation of such enzymes by BCG infections should also be examined. 

Multicolor analysis suggested that BCG infection enhanced the cleavage of OPN and the presence of C-half of OPN determined by 10A16 antibody at apparent extracellular space. We also investigated the amounts of various forms of OPN in CS of the BCG-infected cells. Because of the lack of specific ELISA for C-half OPN and low amounts of FL-OPN in BCG-infected cells, we could not confirm the enhanced cleaved products by any of the three ELISA systems. A very low production of trOPN in BCG-infected cells may indicate their intracellular localization. It was interesting that Ud-OPN was not detected in CS of non-stimulated cells, and a strong positive signal was found only in PMA-treated conditions. It was previously proposed that Ud-OPN contains some cleaved form of OPN [9]. Studies also have shown a negative correlation for Ud-OPN with the number of ESAT-6-specific IFN-γ spot-forming cells in TB patients, but not for FL-OPN or trOPN [10]. These findings indicate that Ud-OPN may have more immune-related functions than does FL-OPN or trOPN. 

We and others have reported elevated levels of OPN, and its association with the severity in MTB infection [7,26]. It is also known that IFN-γ treatment of THP-1 cells induces OPN mRNA levels and protein expression, and it is suggested that OPN may function in a positive-feedback loop during Th1 inflammation [27]. Therefore, it is not clear if infected macrophages or if the cells involved in the immune response against MTB release OPN. Our data from all three assays employed showed that expression of OPN proteins reduced in BCG-infected THP-1 cells. However, concomitant appearance of the cleaved products during BCG infection suggest the degradation of OPN in cells, which might be occurring within the activated lysosome. The difference in localization of positive-staining products is interesting, as seen by intracellular staining by 34E3, and extracellular staining by 10A16. Different interacting molecules of the N-half of OPN (integrin) and the C-half of OPN (CD44 variant) may explain these results [28]. The C-half of OPN may interact with CD44, and this interaction may facilitate the release of the molecule, since it is reported that soluble plasma CD44 levels are closely associated with OPN in adult T-cell lymphoma (ATL) and MTB infection [10,17]. 

It has been reported that lung macrophages that harbor BCG in vivo have decreased MHC-II expression, possibly contributing to BCG evasion from T-cell responses [29]. BCG is the most successful immunotherapeutic agent for high-risk non-muscle invasive bladder cancer [30], and it is also known that the plasma OPN level is associated with the clinical features of bladder cancer [31]. Therefore, the suppression of OPN in macrophages may be related to the anti-bladder-cell carcinoma activity of BCG.

In conclusion, we have characterized various forms of OPN in PMA-stimulated macrophages and have identified a novel, small fragment. Furthermore, BCG infection facilitated the cleavage of OPN and reduced its synthesis. This inhibition may explain the anti-tumor activities of BCG.

## 4. Materials and Methods

### 4.1. Cell Lines and Culture

Human THP-1 monocytic cells derived from acute monocyte leukemia patients [32] were obtained from the American Type Culture Collection (Manassas, VA, USA). Cells were maintained in Roswell Park Memorial Institute (RPMI) 1640 medium (Wako Pure Chemical Industries Ltd., Osaka, Japan). The medium was supplemented with 10% of heat-inactivated fetal bovine serum (FBS) (Thermo Fisher Scientific, Waltham, MA, USA). Cells were cultured at 37 °C in a humidified atmosphere of 5% CO_2_.

### 4.2. BCG Infection

*M. bovis* BCG-Pasteur strain was used in infection assay. One million of THP-1 cells were cultured in 2 mL RPMI medium containing 10% FBS (Biosera, Nuaille, France) (complete medium) in the presence of PMA (10 ng/mL) for 2 days in 35 mm plastic culture dishes at 37 °C at 5% CO_2_ followed by washing three times with phosphate-buffered saline (PBS) (−). BCG infection was conducted as described elsewhere [33]. Washed cells were further cultured for one day in 1.5 mL complete medium. BCG (1.6 × 10^4^ CFU) in 1 mL of complete medium was added to the culture dish for 2 h at 37 °C. Following removal of the medium, the attached cells were washed three times by PBS (−) and the cells were cultured in 1.5 mL complete medium containing gentamycin (GE, 50 μg/mL) for 4 h at 37 °C. After washing twice with PBS (−), cells were cultured in complete medium with GE (10 μg/mL). The infected cells were harvested at days 1, 3 and 4 post infection.

### 4.3. Western Blot

Washed THP-1 cells (5 × 10^5^) were lysed on ice for 15 min using 100 μL of lysis buffer from a WSE-7420 EzRIPA Lysis kit (ATTO Corporation, Tokyo, Japan) and the supernatants were processed by centrifuging at 14,000× *g* for 5–15 min, according to manufacturer’s protocol. The protein concentration was determined by the method of Bradford (Bio-Rad Protein Assay, Hercules, CA, USA). Twenty micrograms of protein from each sample were separated by sodium dodecyl sulfate-polyacrylamide gel electrophoresis (SDS-PAGE) using standard 15% or 5–20% gradient gels, followed by Western blot analysis using an iBlot dry blotting system (Invitrogen, Carlsbad, CA, USA) according to the manufacturer’s instructions. Antibodies O-17 (1:100 dilution; IBL, Gunma, Japan), 34E3 (1:100 dilution; IBL), 10A16 (1:200 dilution; IBL), and ab8448 (1:1000 dilution; Abcam, Tokyo, Japan) were used as primary antibodies (1 μg/mL each). The bands were detected using a WesternBreeze Chromogenic Western Blot Immunodetection Kit (Invitrogen). In some experiments, the bound primary antibodies were reacted with horseradish peroxidase-conjugated mouse or rabbit anti-mouse IgG (Sigma-Aldrich Co. LLC, St. Louis, MO, USA) diluted 1:20,000 and visualized using a TMB Membrane Peroxidase Substrate system (Kirkegaard & Perry Laboratories, Inc., Gaithersburg, MD, USA) according to the manufacturer’s protocol.

### 4.4. Immunofluorescence

#### 4.4.1. Single-Color Analysis

Cells were cultured on polystyrene-treated, 8-well chambered tissue culture glass slides (BD Falcon, Bedord, MA, USA). Cells were rinsed three times in PBS and then incubated for 1 h with secondary antibodies (1:800 dilution) of either goat anti-mouse IgG, or goat anti-rabbit IgG conjugated with Alexa Fluor^®^ 488 (AF488) in PBS containing 3% (*w*/*v*) bovine serum albumin (BSA). After washing the fluorescent-labeled cells, the AF-488 fluorescent tag was excited and detected using a CKX41N-41FL/PHP fluorescence microscope (Olympus, Tokyo, Japan) with filter set B [34]. 

#### 4.4.2. Multicolor Analysis

PMA-stimulated THP-1 cells on 2-well chambered glass slides (Nunc Lab-Tek™, Thermo Fisher Scientific, Waltham, MA, USA) were fixed with 4% (*w*/*v*) paraformaldehyde in PBS (−) for 30 min at 4 °C. The cells were incubated for 1 h with PBS (−) containing 0.3% (*v*/*v*) Triton-X100, 5% (*v*/*v*) normal goat serum and each of the appropriate primary antibodies. The secondary antibodies AF488 and goat anti-mouse IgG (H + L)-Alexa Fluor^®^ 546 (AF546) were purchased from Thermo Fisher Scientific. For nuclear staining, 4′,6-diamidino-2-phenylindole (DAPI) was used. Each of the primary antibodies, the secondary antibodies, and DAPI were diluted to 1 μg/mL with PBS (−) containing 0.3% (*v*/*v*) Triton-X100 and 1% (*w*/*v*) BSA and incubated each for 1 h at 4 °C. Between incubations, the cells were washed twice with PBS (−). The cover glass was applied onto the slide glass. The images were documented with a BZ-X700 fluorescence microscope (Keyence, Osaka, Japan) and quantified using BZ-X analyzer.

### 4.5. ELISAs

Plasma concentrations of OPN were determined using the Human Osteopontin DuoSet ELISA Development System Kit (R&D Systems, Minneapolis, MN, USA) [9]. The proprietary capture monoclonal antibody and the detection polyclonal antibodies in this ELISA kit were both generated against recombinant human OPN (NS0-derived, amino acids Ile17-Asn300). However, the epitopes for these antibodies were not disclosed.

To identify the full-length OPN and trOPN, two separate ELISA kits (IBL, Gunma, Japan) were used [9]. In the FL-OPN kit, O-17, a polyclonal rabbit antibody specific to the N-terminus of OPN was used as a capture antibody, and mouse monoclonal antibody 10A16 served as a detector antibody. The trOPN ELISA assay was performed using 34E3, an anti-trOPN monoclonal antibody, as the capture antibody, and the O-17 antibody as the detection antibody. This capture antibody specifically reacts to the Ser162–Arg168 epitope.

### 4.6. Statistical Analysis

Unpaired *t*-tests (two-tailed) were used to compare the results obtained from the BCG-infected and mock-infected. The values of *p* < 0.05 were considered statistically significant. All statistical analyses were performed using GraphPad Prism software, version 6 (GraphPad Software Inc., San Diego, CA, USA). 

## 5. Conclusions

Following PMA stimulation of human monocytic THP-1 cells, a small novel protein was detected with antibody 34E3 and a protease-cleaved epitope was found. Antibody 34E3 also bound to BCG-infected cells. ELISA showed that the release of OPNs was inhibited by BCG infection. The N-half and C-half of cleaved OPN was found in BCG-infected THP-1 cells, and the product may play an important role in memory T-cell migration in infectious diseases.

## Figures and Tables

**Table 1:** OPNs in supernatants of PMA-stimulated THP-1 cells.

Reagents	FL-OPN ^2^	trOPN ^3^	Ud-OPN ^4^
PMA (−)	2509 ± 56.1	12.4 ± 0.85	1.40 ± 0.22
^1^ PMA (+)	10656 ± 869	17.9 ± 6.30	76.2 ± 12.1

^1^ THP-1 cells were cultured for two days with PMA at 10 ng/mL. ^2^ Full-length OPN. ^3^ Thrombin-cleaved OPN. ^4^ Undefined-OPN.

## Abbreviations

OPN	Osteopontin
FL-OPN	Full-length OPN
Tr-OPN	Thrombin-cleaved form of OPN
Ud-OPN	Undefined OPN
PMA	Phorbol 12-myristate 13-acetate
